# The genome sequence of the Heterolobosean amoeboflagellate, *Tetramitus jugosus *CCAP 1588/3C

**DOI:** 10.12688/wellcomeopenres.20189.1

**Published:** 2023-11-10

**Authors:** David H. Green, Cecilia Rad-Menéndez, Culture Collection of Algae and Protozoa collective, Earlham Institute Genome Acquisition Lab and Protists Project, University of Oxford and Wytham Woods Genome Acquisition Lab, Darwin Tree of Life Barcoding collective, Wellcome Sanger Institute Tree of Life programme, Wellcome Sanger Institute Scientific Operations: DNA Pipelines collective, Tree of Life Core Informatics collective, Darwin Tree of Life Consortium

**Affiliations:** 1 Culture Collection of Algae and Protozoa, The Scottish Association for Marine Science, Oban, Scotland, UK

**Keywords:** Tetramitus jugosus, amoeboflagellate, genome sequence, chromosomal, Heterolobosea, Schizopyrenida

## Abstract

We present a genome assembly from cultivated *Tetramitus jugosus* (Heterolobosea; Schizopyrenida; Vahlkampfiidae). The genome sequence is 26.3 megabases in span. Most of the assembly (99.3%) is scaffolded into 52 chromosomal pseudomolecules. The mitochondrial genome has also been assembled and is 49.46 kilobases in length.

## Species taxonomy

Eukaryota; Discoba; Heterolobosea; Tetramitia; Eutetramitia; Vahlkampfiidae; *Tetramitus; Tetramitus jugosus* CCAP 1588/3C (NCBI:txid166959).

## Background

*Tetramitus jugosus* CCAP 1588/3C ( Brown & de Jonckheere, 1999) is an Heterolobosean amoeboflagellate characterised by movement through pseudopodia, intranuclear promitosis, flattened mitochondrial cristae and an absence of stacked Golgi bodies ( Page & Blanton, 1985). *Tetramitus* belongs to the family Vahlkampfiidae, and was originally established as a new genus, *Paratretamitus*, due to its capacity to transform into flagellated stages ( Darbyshire *et al.*, 1976), however, it was later transferred to the genus *Tetramitus* following the sequencing of its small subunit ribosomal DNA (SSU rDNA) ( Brown & de Jonckheere, 1999; de Jonckheere *et al*., 2011).

**Figure 1.**
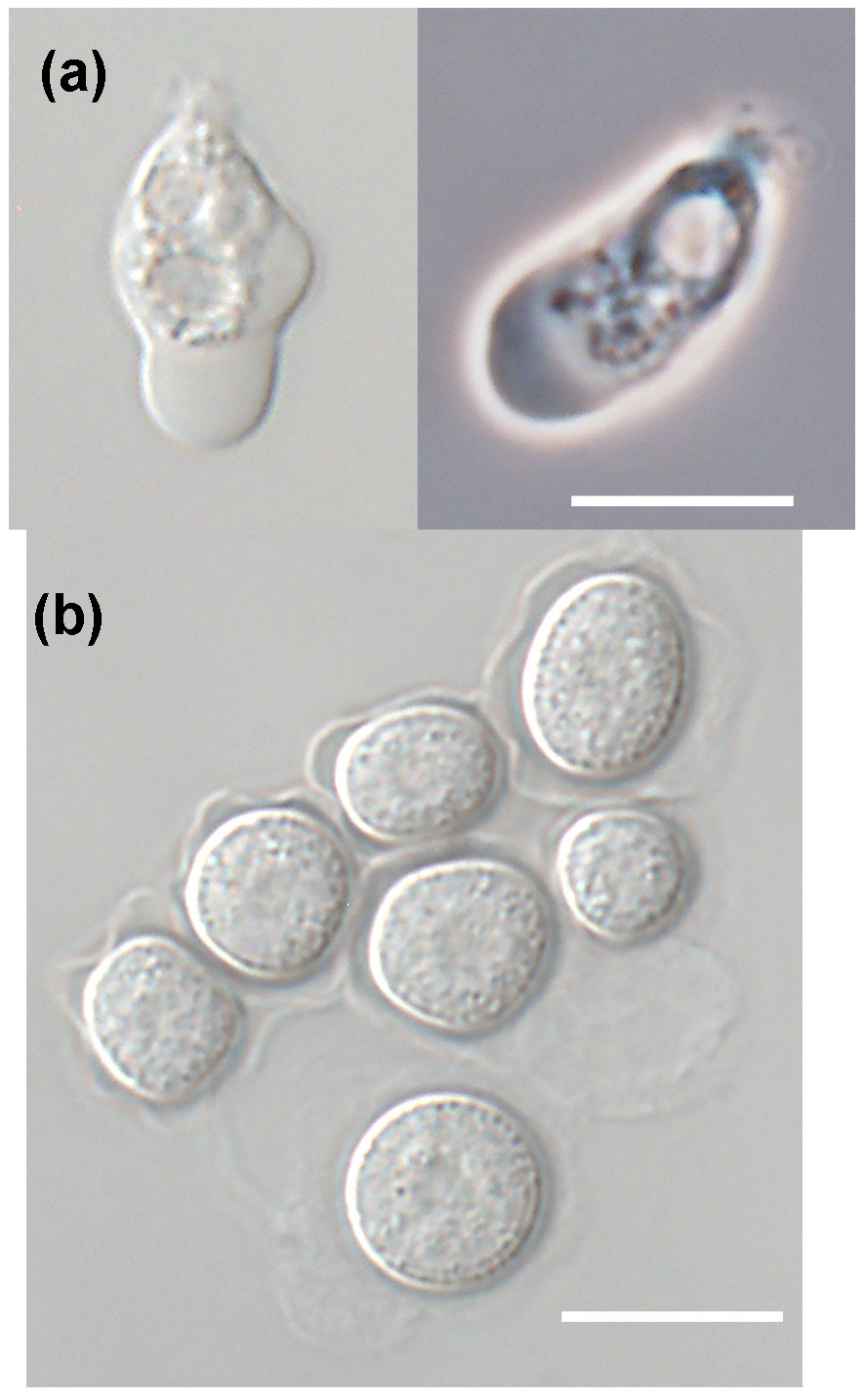
Amoeboid ( **a**) and cyst ( **b**) lifecycle stages of *T. jugosus*. Scale bar, 10 µm.

*T. jugosus* can shift between an amoeboid vegetative state and flagellated form ( Figure 1a), and, under unfavourable environmental conditions, it can form dormant cysts ( Figure 1b) to survive in the environment. Once the conditions become favourable, they can excyst back to the ameboid form. *Tetramitus* feeds on bacteria, fungi, or other protists, engulfing them by phagocytosis into digestive vacuoles.

The Culture Collection of Algae and Protozoa *T. jugosus* strain (CCAP 1588/3C) was isolated in 1974 from soil collected at a depth of 5–15 cm in Morayshire, Scotland. The strain is maintained as a living culture and successfully cryopreserved at the CCAP, which ensures long-term safekeeping and open access to viable cultures of this organism.

*Tetramitus* are important members of soil microbial communities and predators of bacteria that play a key ecological role regulating microbial populations in the environment and therefore promoting plant growth ( Bonkowski, 2004; Rosenberg *et al.*, 2009).

The genome of *T. jugosus* CCAP 1588/3C will help address a grand challenge in protists research, namely the lack of relevant genome sequences of important protozoa species.

## Genome sequence report

The genome was sequenced from a sample of cultivated cells of *T. jugosus* (paTetJugo1). A total of 37-fold coverage in Pacific Biosciences single-molecule HiFi long was generated. Primary assembly contigs were scaffolded with chromosome conformation Hi-C data. Manual assembly curation corrected 7 missing or misjoins and removed 7 haplotypic duplications, reducing the assembly length by 7.91% and the scaffold number by 1.79%, and decreasing the scaffold N50 by 5.57%.

**Figure 2.**
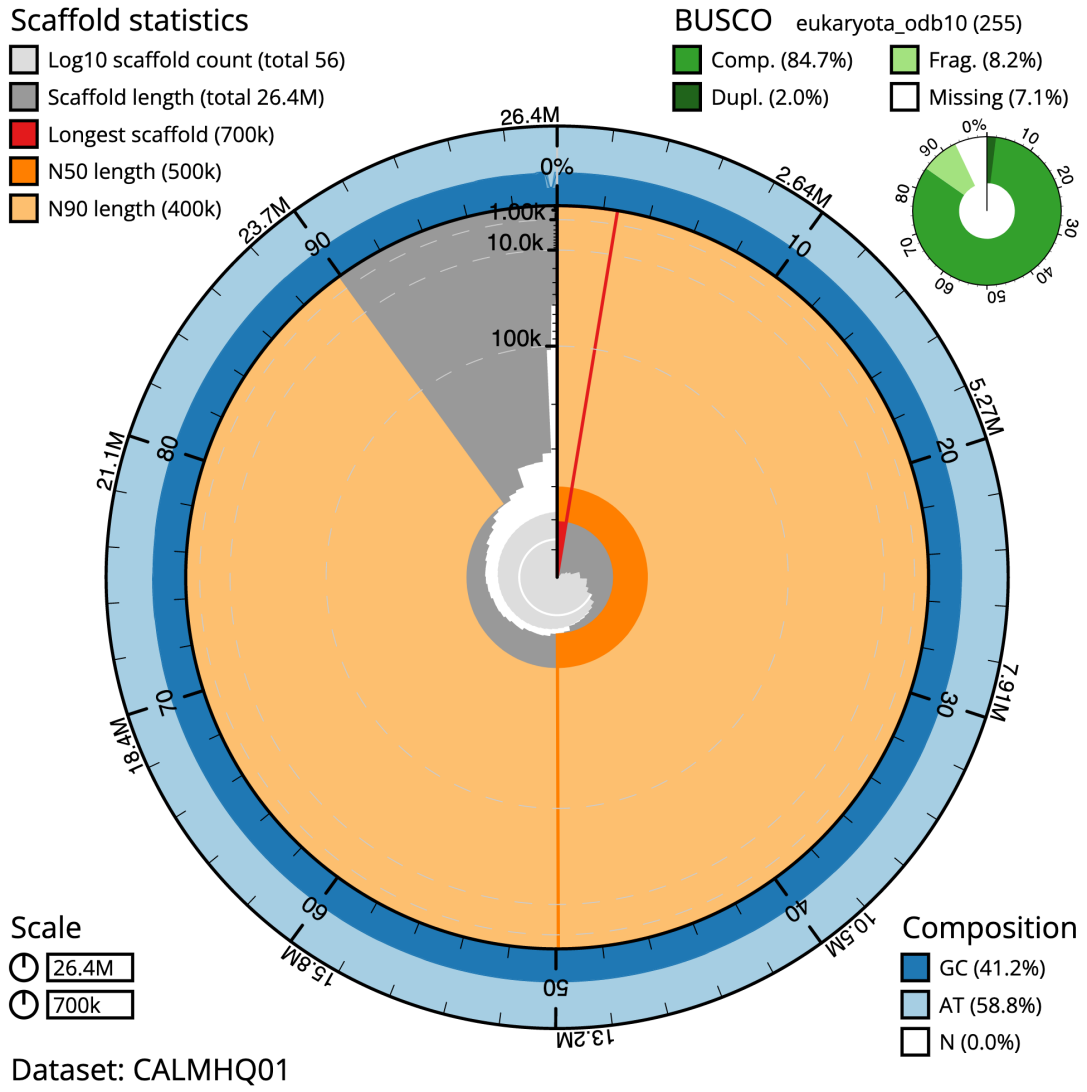
Genome assembly of *T. jugosus*, paTetJugo1.1: metrics. The BlobToolKit Snailplot shows N50 metrics and BUSCO gene completeness. The main plot is divided into 1,000 size-ordered bins around the circumference with each bin representing 0.1% of the 26,350,207 bp assembly. The distribution of chromosome lengths is shown in dark grey with the plot radius scaled to the longest chromosome present in the assembly (699,783 bp, shown in red). Orange and pale-orange arcs show the N50 and N90 chromosome lengths (499,551 and 399,693 bp), respectively. The pale grey spiral shows the cumulative chromosome count on a log scale with white scale lines showing successive orders of magnitude. The blue and pale-blue area around the outside of the plot shows the distribution of GC, AT and N percentages in the same bins as the inner plot. A summary of complete, fragmented, duplicated and missing BUSCO genes in the eukaryota_odb10 set is shown in the top right. An interactive version of this figure is available at https://blobtoolkit.genomehubs.org/view/paTetJugo1.1/dataset/CALMHQ01/snail.

**Figure 3.**
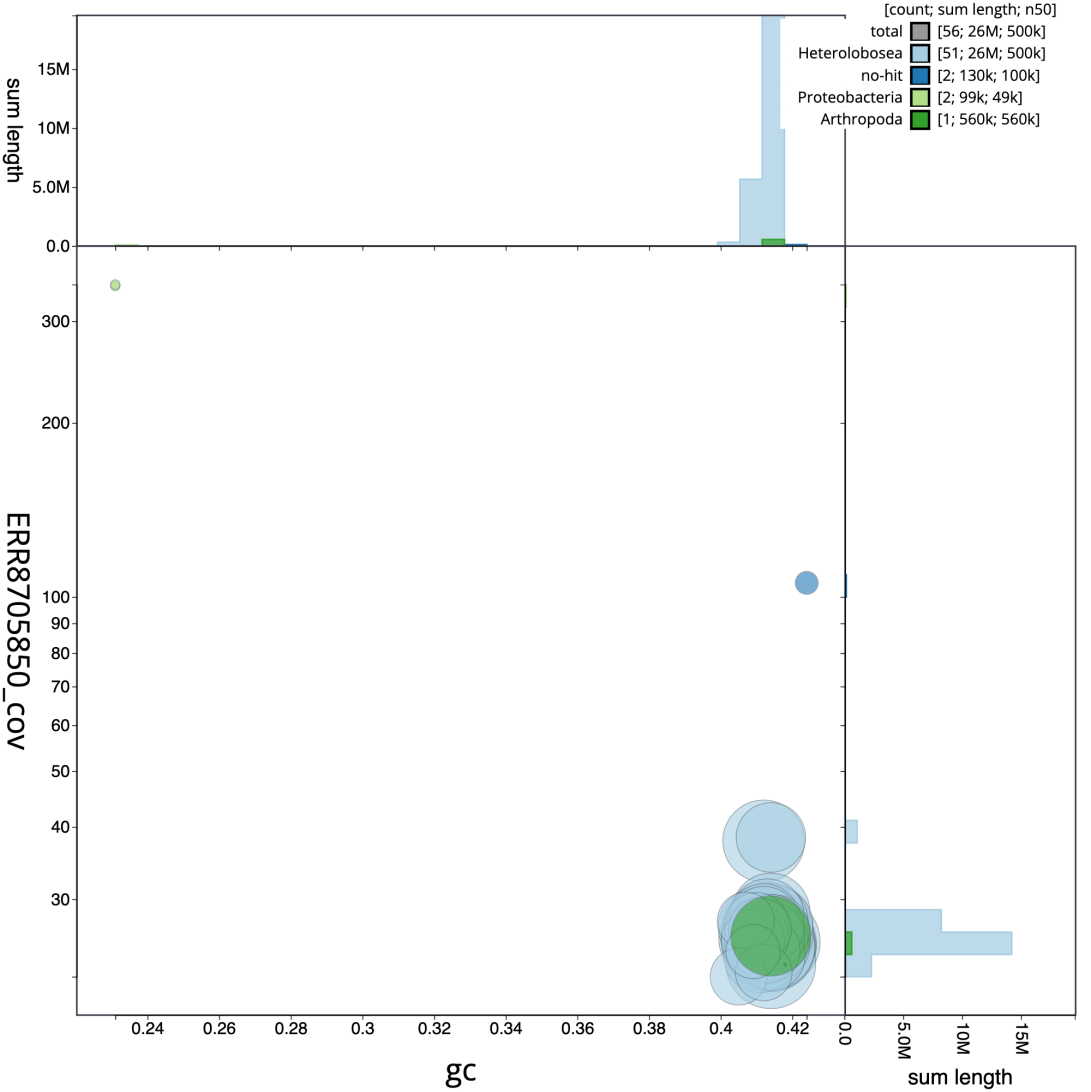
Genome assembly of *T. jugosus*, paTetJugo1.1: GC coverage. BlobToolKit GC-coverage plot. Chromosomes are coloured by phylum. Circles are sized in proportion to chromosome length. Histograms show the distribution of chromosome length sum along each axis. An interactive version of this figure is available at https://blobtoolkit.genomehubs.org/view/paTetJugo1.1/dataset/CALMHQ01/blob.

**Figure 4.**
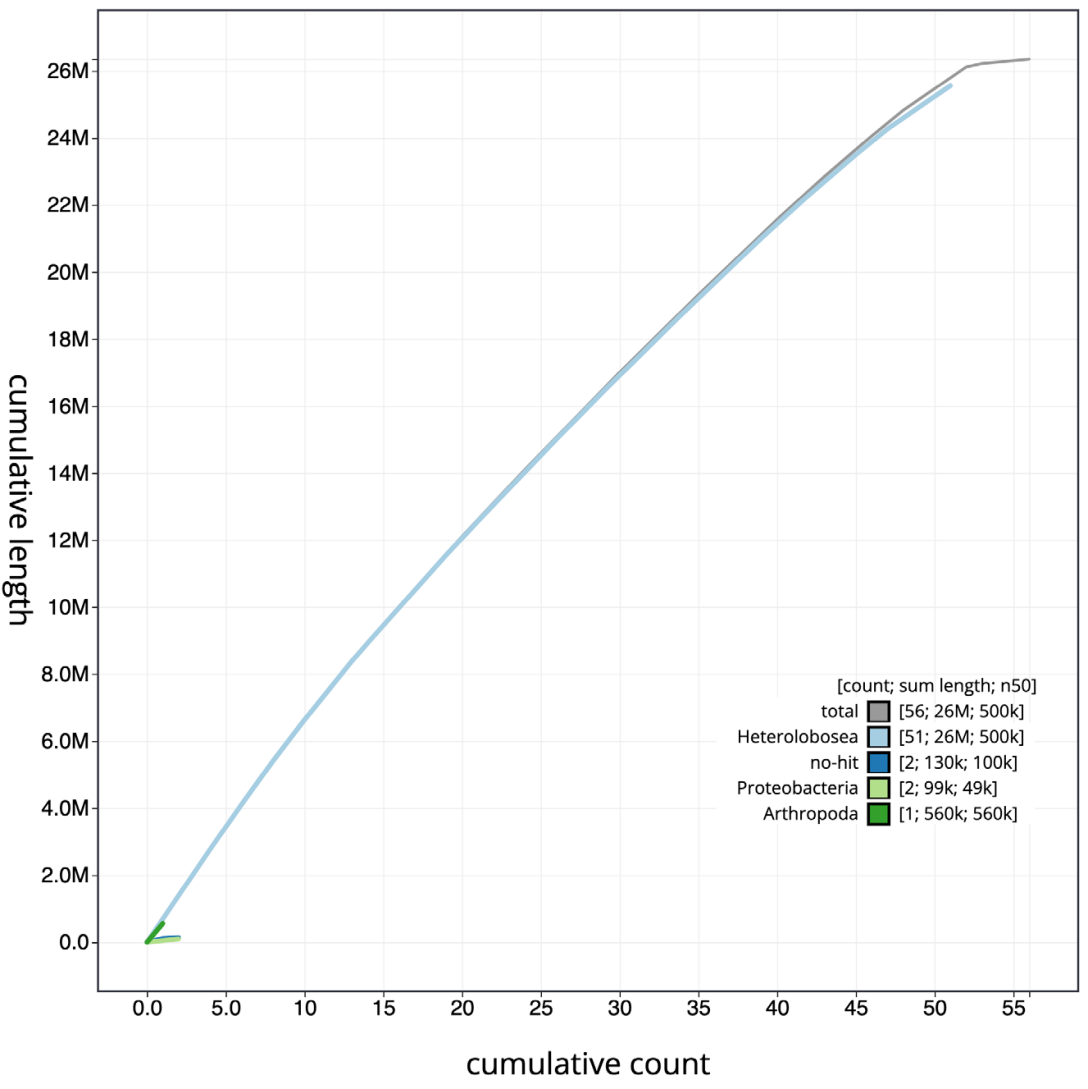
Genome assembly of *T. jugosus*, paTetJugo1.1: cumulative sequence. BlobToolKit cumulative sequence plot. The grey line shows cumulative length for all chromosomes. Coloured lines show cumulative lengths of chromosomes assigned to each phylum using the buscogenes taxrule. An interactive version of this figure is available at https://blobtoolkit.genomehubs.org/view/paTetJugo1.1/dataset/CALMHQ01/cumulative.

**Figure 5.**
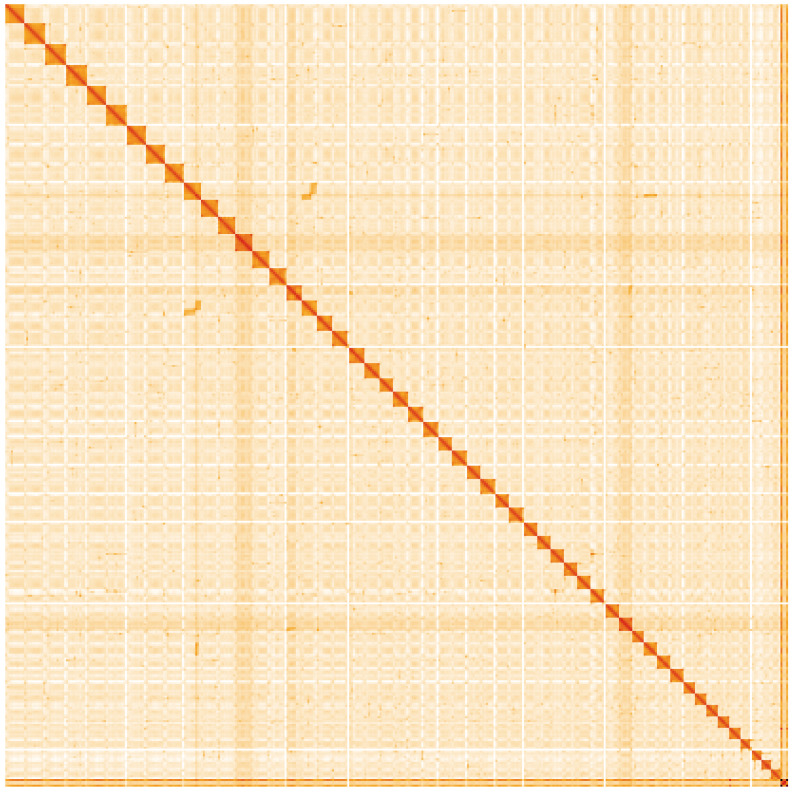
Genome assembly of *Tetramitus jugosus*, paTetJugo1.1: Hi-C contact map of the paTetJugo1.1 assembly, visualised using HiGlass. Chromosomes are shown in order of size from left to right and top to bottom. An interactive version of this figure may be viewed at https://genome-note-higlass.tol.sanger.ac.uk/l/?d=LpwbNNzHQsO6-sVUyCcfRQ.

The final assembly has a total length of 26.3 Mb in 55 sequence scaffolds with a scaffold N50 of 0.5 Mb ( Table 1). A summary of the assembly statistics is shown in Figure 2, while the distribution of assembly scaffolds on GC proportion and coverage is shown in Figure 3. The cumulative assembly plot in Figure 4 shows curves for subsets of scaffolds assigned to different phyla. Most (99.3%) of the assembly sequence was assigned to 52 chromosomal-level scaffolds. Chromosome-scale scaffolds confirmed by the Hi-C data are named in order of size ( Figure 5; Table 2). While not fully phased, the assembly deposited is of one haplotype. Contigs corresponding to the second haplotype have also been deposited. The mitochondrial genome was also assembled and can be found as a contig within the multifasta file of the genome submission.

**Table 1.** Genome data for *Tetramitus jugosus*, paTetJugo1.1.

Project accession data		
Assembly identifier	paTetJugo1.1	
Species	*Tetramitus jugosus*	
Specimen	paTetJugo1 CCAP 1588/3C	
NCBI taxonomy ID	166959	
BioProject	PRJEB50460	
BioSample ID	SAMEA7524615	
Isolate information	paTetJugo1, cultivated cells	
Assembly metrics *		*Benchmark*
Consensus quality (QV)	56.7	*≥ 50*
*k*-mer completeness	100%	*≥ 95%*
BUSCO **	C:84.7%[S:82.7%,D:2.0%],F:8.2%,M:7.1%,n:255	*C ≥ 95%*
Percentage of assembly mapped to chromosomes	99.3%	*≥ 95%*
Sex chromosomes	-	*localised homologous pairs*
Organelles	Mitochondrial genome assembled	*complete single alleles*
Raw data accessions		
PacificBiosciences SEQUEL II	ERR8705850, ERR8705851	
Hi-C Illumina	ERR8571633	
Genome assembly		
Assembly accession	GCA_937625935.1	
*Accession of alternate haplotype*	GCA_937658345.1	
Span (Mb)	26.3	
Number of contigs	57	
Contig N50 length (Mb)	0.5	
Number of scaffolds	55	
Scaffold N50 length (Mb)	0.5	
Longest scaffold (Mb)	0.7	

* Assembly metric benchmarks are adapted from column VGP-2020 of “Table 1: Proposed standards and metrics for defining genome assembly quality” from ( Rhie *et al.*, 2021). ** BUSCO scores based on the eukaryota_odb10 BUSCO set using v5.3.2. C = complete [S = single copy, D = duplicated], F = fragmented, M = missing, n = number of orthologues in comparison. A full set of BUSCO scores is available at https://blobtoolkit.genomehubs.org/view/paTetJugo1.1/dataset/CALMHQ01/busco.

**Table 2.** Chromosomal pseudomolecules in the genome assembly of *Tetramitus jugosus*, paTetJugo1.

INSDC accession	Chromosome	Size (Mb)	GC%
OW569344.1	1	0.7	41.3
OW569345.1	2	0.69	41.3
OW569346.1	3	0.69	41.5
OW569347.1	4	0.69	41.4
OW569348.1	5	0.67	41.4
OW569349.1	6	0.67	41.4
OW569350.1	7	0.66	41.4
OW569351.1	8	0.64	41.3
OW569352.1	9	0.61	41.3
OW569353.1	10	0.61	41.4
OW569354.1	11	0.59	41.1
OW569355.1	12	0.59	41.3
OW569356.1	13	0.58	41.2
OW569357.1	14	0.56	41.4
OW569358.1	15	0.54	41.3
OW569359.1	16	0.53	41.4
OW569360.1	17	0.53	41.2
OW569361.1	18	0.53	41.2
OW569362.1	19	0.52	41
OW569363.1	20	0.52	41.5
OW569364.1	21	0.51	41.3
OW569365.1	22	0.5	41.3
OW569366.1	23	0.5	41.4
OW569367.1	24	0.49	41.3
OW569368.1	25	0.49	41.4
OW569369.1	26	0.49	41.1
OW569370.1	27	0.49	41.3
OW569371.1	28	0.48	41.1
OW569372.1	29	0.48	41.2
OW569373.1	30	0.47	41.2
OW569374.1	31	0.47	41.3
OW569375.1	32	0.47	41.1
OW569376.1	33	0.47	41.1
OW569377.1	34	0.46	41.2
OW569378.1	35	0.46	41
OW569379.1	36	0.45	41.5
OW569380.1	37	0.45	41.1
OW569381.1	38	0.45	41.5
OW569382.1	39	0.45	41.4
OW569383.1	40	0.44	41.3
OW569384.1	41	0.43	41.4
OW569385.1	42	0.43	41.3
OW569386.1	43	0.42	41.4
OW569387.1	44	0.41	41.3
OW569388.1	45	0.4	41
OW569389.1	46	0.4	41
OW569390.1	47	0.39	40.8
OW569391.1	48	0.38	41.1
OW569392.1	49	0.33	40.7
OW569393.1	50	0.33	40.5
OW569394.1	51	0.33	41.2
OW569395.1	52	0.31	40.9
OW569396.1	MT	0.05	23.1

The estimated Quality Value (QV) of the final assembly is 56.7 with *k*-mer completeness of 100%, and the assembly has a BUSCO v5.3.2 completeness of 84.7% (single = 82.7%, duplicated = 2.0%), using the eukaryota_odb10 reference set ( *n* = 255).

Metadata for specimens, spectral estimates, sequencing runs, contaminants and pre-curation assembly statistics can be found at https://links.tol.sanger.ac.uk/species/166959.

## Methods

### Sample acquisition and nucleic acid extraction

*T. jugosus* CCAP 1588/3C was cultivated in Non-Nutrient agar ( Page, 1976) streaked with a non-pathogenic strain of *Escherichia coli* as food source. The strain was incubated at 20°C with no direct light ca. 5 µmol m ^–1^ s ^–1^ for a period of 12:12 h light:dark. Once all the *E. coli* was consumed, the biomass was harvested by washing the cells from the agar with sterile Page’s Amoeba Saline Solution ( Page, 1976; Page, 1988) and pelleted by centrifugation. The pellet was snap frozen in liquid nitrogen, the samples were then stored at –80°C and shipped on dry ice.

DNA was extracted at the Tree of Life laboratory, Wellcome Sanger Institute. The paTetJugo1 sample was weighed and some of the sample was set aside for Hi-C sequencing. The cells were cryogenically disrupted to a fine powder using a Covaris cryoPREP Automated Dry Pulveriser, receiving multiple impacts. Fragment size analysis of 0.01-0.5 ng of DNA was then performed using an Agilent FemtoPulse. High molecular weight (HMW) DNA was extracted using the Qiagen MagAttract HMW DNA extraction kit. Low molecular weight DNA was removed from a 20 ng aliquot of extracted DNA using 0.8X AMpure XP purification kit prior to sequencing; a minimum of 50 ng DNA was submitted for sequencing. HMW DNA was sheared into an average fragment size of 12–20 kb in a Megaruptor 3 system with speed setting 30. Sheared DNA was purified by solid-phase reversible immobilisation using AMPure PB beads with a 1.8X ratio of beads to sample to remove the shorter fragments and concentrate the DNA sample. The concentration of the sheared and purified DNA was assessed using a Nanodrop spectrophotometer and Qubit Fluorometer and Qubit dsDNA High Sensitivity Assay kit. Fragment size distribution was evaluated by running the sample on the FemtoPulse system.

### Sequencing

Pacific Biosciences HiFi circular consensus DNA sequencing libraries were constructed according to the manufacturers’ instructions. DNA sequencing was performed by the Scientific Operations core at the WSI on a Pacific Biosciences SEQUEL II (HiFi) instrument. Hi-C data were also generated from a sample of paTetJugo1 using the Arima2 kit and sequenced on the Illumina NovaSeq 6000 instrument.

### Genome assembly, curation and evaluation

The assembly process included the following sequence of steps: Tiara 1.0.1 ( Karlicki *et al.*, 2022) was run with PacBio HiFi reads. Reads classified as prokaryotic by Tiara were removed and the remaining reads were assembled using Hifiasm ( Cheng *et al.*, 2021) with default settings. The Hifiasm contigs were checked for contaminants using Tiara 1.0.1 and BLAST against NCBI nt and nr databases (May 2021 versions). Mitochondrial and plastid contigs were detected using Tiara. The longest organellar contigs were circularised using circlator minimus2 (1.5.5) ( Hunt *et al.*, 2015). The remaining copies of organellar contigs were discarded. Deduplication of chromosomal contigs was done using GAP5 (1.2.14-r) ( Bonfield & Whitwham, 2010). The assemblies were scaffolded with Hi-C Arima2 data using SALSA2 ( Ghurye *et al.*, 2019). The assembly was checked for contamination and corrected using the gEVAL system ( Chow *et al.*, 2016) as described previously ( Howe *et al.*, 2021). Manual curation was performed using gEVAL, HiGlass ( Kerpedjiev *et al.*, 2018) and Pretext ( Harry, 2022).

A Hi-C map for the final assembly was produced using bwa-mem2 ( Vasimuddin *et al.*, 2019) in the Cooler file format ( Abdennur & Mirny, 2020). To assess the assembly metrics, the *k*-mer completeness and QV consensus quality values were calculated in Merqury ( Rhie *et al.*, 2020). This work was done using Nextflow ( Di Tommaso *et al.*, 2017) DSL2 pipelines “sanger-tol/readmapping” ( Surana *et al.*, 2023a) and “sanger-tol/genomenote” ( Surana *et al.*, 2023b). The genome was analysed within the BlobToolKit environment ( Challis *et al.*, 2020) and BUSCO scores ( Manni *et al.*, 2021; Simão *et al.*, 2015) were calculated.

Table 3 contains a list of relevant software tool versions and sources.

**Table 3.** Software tools: versions and sources.

Software tool	Version	Source
BlobToolKit	4.0.7	https://github.com/blobtoolkit/blobtoolkit
BUSCO	5.3.2	https://gitlab.com/ezlab/busco
circlator minimus2	1.5.5	https://github.com/sanger-pathogens/circlator
gEVAL	N/A	https://geval.org.uk/
Hifiasm	0.12	https://github.com/chhylp123/hifiasm
HiGlass	1.11.6	https://github.com/higlass/higlass
Merqury	MerquryFK	https://github.com/thegenemyers/MERQURY.FK
MitoHiFi	2	https://github.com/marcelauliano/MitoHiFi
PretextView	0.2	https://github.com/wtsi-hpag/PretextView
purge_dups	1.2.3	https://github.com/dfguan/purge_dups
SALSA	2.2	https://github.com/salsa-rs/salsa
sanger-tol/genomenote	v1.0	https://github.com/sanger-tol/genomenote
sanger-tol/readmapping	1.1.0	https://github.com/sanger-tol/readmapping/tree/1.1.0
Tiara	1.0.1	https://github.com/ibe-uw/tiara

### Wellcome Sanger Institute – Legal and Governance

The materials that have contributed to this genome note have been supplied by a Darwin Tree of Life Partner. The submission of materials by a Darwin Tree of Life Partner is subject to the **‘Darwin Tree of Life Project Sampling Code of Practice’**, which can be found in full on the Darwin Tree of Life website here. By agreeing with and signing up to the Sampling Code of Practice, the Darwin Tree of Life Partner agrees they will meet the legal and ethical requirements and standards set out within this document in respect of all samples acquired for, and supplied to, the Darwin Tree of Life Project. 

Further, the Wellcome Sanger Institute employs a process whereby due diligence is carried out proportionate to the nature of the materials themselves, and the circumstances under which they have been/are to be collected and provided for use. The purpose of this is to address and mitigate any potential legal and/or ethical implications of receipt and use of the materials as part of the research project, and to ensure that in doing so we align with best practice wherever possible. The overarching areas of consideration are:

•  Ethical review of provenance and sourcing of the material

•  Legality of collection, transfer and use (national and international) 

Each transfer of samples is further undertaken according to a Research Collaboration Agreement or Material Transfer Agreement entered into by the Darwin Tree of Life Partner, Genome Research Limited (operating as the Wellcome Sanger Institute), and in some circumstances other Darwin Tree of Life collaborators.

## Data Availability

European Nucleotide Archive: *Tetramitus jugosus*. Accession number PRJEB50460; https://identifiers.org/ena.embl/PRJEB50460. ( Wellcome Sanger Institute, 2022) The genome sequence is released openly for reuse. The *Tetramitus jugosus* genome sequencing initiative is part of the Darwin Tree of Life (DToL) project. All raw sequence data and the assembly have been deposited in INSDC databases. The genome will be annotated using available RNA-Seq data and presented through the Ensembl pipeline at the European Bioinformatics Institute. Raw data and assembly accession identifiers are reported in Table 1.

## Author information

Members of the Earlham Institute Genome Acquisition Lab and Protists Project are given here: https://doi.org/10.5281/zenodo.8395242.

Members of the Culture Collection of Algae & Protozoa are given here: https://doi.org/10.5281/zenodo.7650573.

Members of the University of Oxford and Wytham Woods Genome Acquisition Lab are listed here: https://doi.org/10.5281/zenodo.4789928.

Members of the Darwin Tree of Life Barcoding collective are listed here: https://doi.org/10.5281/zenodo.4893703.

Members of the Wellcome Sanger Institute Tree of Life programme are listed here: https://doi.org/10.5281/zenodo.4783585.

Members of Wellcome Sanger Institute Scientific Operations: DNA Pipelines collective are listed here: https://doi.org/10.5281/zenodo.4790455.

Members of the Tree of Life Core Informatics collective are listed here: https://doi.org/10.5281/zenodo.5013541.

Members of the Darwin Tree of Life Consortium are listed here: https://doi.org/10.5281/zenodo.4783558.
