# Supplementary material for: The genome sequence of the Heterolobosean amoeboflagellate, Tetramitus jugosus CCAP 1588/3C
Source: Wellcome Open Res. 2023 Nov 10;8:513. [Version 1] doi: 10.12688/wellcomeopenres.20189.1 (PMC11106597; doi:10.12688/wellcomeopenres.20189.1)
Supplement: Peer review history

# Reviewer report: Reviewer response for version 1

**DOI:** 10.21956/wellcomeopenres.22352.r80994

**Published:** 2024-05-22

**Authors:** Oleg N. Shchepin

**Affiliations:** 1 University Greifswald, Greifswald, Germany

The authors provide a high-quality assembly of the nuclear and mitochondrial genomes of *Tetramitus jugosus.* The resulting data are clearly described and are available from the ENA. These data will be valuable for further studies of the phylogeny and evolution of Heterolobosea. For the sake of reproducibility, it would be beneficial for the readers if the authors provided a more in-depth description of the methods of genome assembly and curation, either in the form of the analysis script or specifying the parameter settings for the software they used.

Are sufficient details of methods and materials provided to allow replication by others?

Partly

Is the rationale for creating the dataset(s) clearly described?

Yes

Are the datasets clearly presented in a useable and accessible format?

Yes

Are the protocols appropriate and is the work technically sound?

Yes

Reviewer Expertise:

protistology, phylogenetics, metagenomics

I confirm that I have read this submission and believe that I have an appropriate level of expertise to confirm that it is of an acceptable scientific standard.

# Reviewer report: Reviewer response for version 1

**DOI:** 10.21956/wellcomeopenres.22352.r80992

**Published:** 2024-05-20

**Authors:** Jeremy Wideman

**Affiliations:** 1 Arizona State University, Arizona, USA

The data note presents an excellent assembly of a heterolobosean, *Tetramitus jugosus*. The data appear solid and the methods robust. These data will be instrumental in future comparative analyses as they appear to represent 52 chromosome-length contigs. The methods and data provide a template and standard for future sequencing efforts in this clade.

Are sufficient details of methods and materials provided to allow replication by others?

Yes

Is the rationale for creating the dataset(s) clearly described?

Yes

Are the datasets clearly presented in a useable and accessible format?

Yes

Are the protocols appropriate and is the work technically sound?

Yes

Reviewer Expertise:

Comparative genomics, protistology, evolutionary cell biology

I confirm that I have read this submission and believe that I have an appropriate level of expertise to confirm that it is of an acceptable scientific standard.

# Reviewer report: Reviewer response for version 1

**DOI:** 10.21956/wellcomeopenres.22352.r77982

**Published:** 2024-04-26

**Authors:** Alexander K. Tice

**Affiliations:** 1 Biological Sciences, Texas Tech University (Ringgold ID: 6177), Lubbock, Texas, USA

The authors present the first nuclear and mitochondrial genome assemblies for the free-living heterolobsean amoeboflagellate *Tetramitus jugosus*. Through generation of long-read and conformational Hi-C sequence data the authors were able to produce a highly contiguous and complete nuclear genome along with a circularized mitochondrial genome. These data are a valuable contribution to the comparative genomics community as there are currently only a few publicly available genomes from heterolobosean taxa outside of the genus *Naeglaria*. The authors have made all the raw reads as well as the assemblies themselves publicly available, but the full annotation of the nuclear genome is still in progress. A few welcome additions/clarifications to the manuscript would be 1) any explanation as to the presence of the chromosome assigned to the phylum Arthropoda in the final assembly and 2) more detail on the specific flags and parameter choices in the many software packages used to produce the final assembly.

Are sufficient details of methods and materials provided to allow replication by others?

Partly

Is the rationale for creating the dataset(s) clearly described?

Yes

Are the datasets clearly presented in a useable and accessible format?

Yes

Are the protocols appropriate and is the work technically sound?

Yes

Reviewer Expertise:

protistology, comparative genomics, phylogenetics

I confirm that I have read this submission and believe that I have an appropriate level of expertise to confirm that it is of an acceptable scientific standard.
